# Structural and biochemical characterisation of *Archaeoglobus fulgidus* esterase reveals a bound CoA molecule in the vicinity of the active site

**DOI:** 10.1038/srep25542

**Published:** 2016-05-10

**Authors:** Christopher Sayer, William Finnigan, Michail N. Isupov, Mark Levisson, Servé W. M. Kengen, John van der Oost, Nicholas J. Harmer, Jennifer A. Littlechild

**Affiliations:** 1 The Henry Wellcome Building for Biocatalysis, Biosciences, University of Exeter, Stocker Road, Exeter EX4 4QD, UK; 2 Laboratory of Microbiology, Wageningen University, Stippeneng 4, 6708WE, Wageningen, The Netherlands

## Abstract

A new carboxyl esterase, AF-Est2, from the hyperthermophilic archaeon *Archaeoglobus fulgidus* has been cloned, over-expressed in *Escherichia coli* and biochemically and structurally characterized. The enzyme has high activity towards short- to medium-chain *p*-nitrophenyl carboxylic esters with optimal activity towards the valerate ester. The AF-Est2 has good solvent and pH stability and is very thermostable, showing no loss of activity after incubation for 30 min at 80 °C. The 1.4 Å resolution crystal structure of AF-Est2 reveals Coenzyme A (CoA) bound in the vicinity of the active site. Despite the presence of CoA bound to the AF-Est2 this enzyme has no CoA thioesterase activity. The pantetheine group of CoA partially obstructs the active site alcohol pocket suggesting that this ligand has a role in regulation of the enzyme activity. A comparison with closely related α/β hydrolase fold enzyme structures shows that the AF-Est2 has unique structural features that allow CoA binding. A comparison of the structure of AF-Est2 with the human carboxyl esterase 1, which has CoA thioesterase activity, reveals that CoA is bound to different parts of the core domain in these two enzymes and approaches the active site from opposite directions.

*Archaeoglobus fulgidus* is an anaerobic heterotrophic sulfate reducing archaeon^1^ that grows at temperatures between 60 and 95 °C, with optimal growth at 83 °C. The enzymes from thermophilic microorganisms have high potential for use as new industrial biocatalysts due to their naturally high stability to temperature and organic solvents^2^,^3^.

The esterases are a class of enzymes that catalyse the cleavage of ester bonds and have been extensively used in many important biotechnology applications^4^. These include the synthesis of optically pure compounds in the agriculture, food and pharmaceutical industries^5^ and the degradation of natural materials and pollutants, plastics and other industrial waste products^6^,^7^. Features such as high stability in organic solvents^8^, broad substrate specificity^9^ and high regio- and stereo-selectivity^10^ make these enzymes attractive biocatalysts. Specific examples include the use of aryl esterases in the development of flavours in the food and beverage industries^11^, and the use of the *Bacillus* carboxyl esterase NP for the production of the nonsteroidal drug naproxen^12^.

The lipolytic enzymes are made up from two main groups^13^: the true lipases (EC 3.1.1.3; triacylglycerol lipases) and the esterases (EC 3.1.1.1; carboxylesterases). Enzymes of both groups catalyse the cleavage of an ester into a carboxylic acid and an alcohol. The esterases hydrolyse water soluble esters with a short fatty acid chain, while lipases show preference for insoluble fatty acid esters with a chain length greater than ten carbon units. The esterases obey classical Michaelis-Menten kinetics and have a relatively open active site. In contrast, the lipases use the process of interfacial activation where a hydrophobic lid domain moves away from the active site in the presence of a minimum concentration of a lipid substrate. Most lipolytic enzymes belong to the α/β hydrolase protein fold superfamily. However, esterase activity has been reported for enzymes with a β lactamase fold^14^ and an α/β/α hydrolase fold^15^ or as a side activity for the carbonic anhydrase enzymes^16^. The ESTHER database^17^ divides the α/β hydrolase enzymes into over 140 families and superfamilies which are further assigned to groups C, H, L, and X.

The proposed mechanism of catalysis by the α/β hydrolase lipolytic enzymes resembles the serine protease mechanism^18^. This involves the substrate binding with the carbonyl oxygen adjacent to the oxyanion hole. The catalytic acidic residues and histidine activate the catalytic serine hydroxyl which performs nucleophilic attack on the carbonyl carbon of the scissile bond to produce the tetrahedral intermediate. Subsequently the alcohol product is released and an acyl-enzyme complex is formed. After an attack by a water molecule another tetrahedral intermediate is formed which resolves to release the carboxyl product and the free enzyme. The catalytic serine residue in the α/β hydrolase fold esterases is usually located in a tight nucleophilic elbow with the consensus sequence Gly-X-Ser-X-Gly, although deviations from this consensus have been reported^19^.

Many examples of thermophilic esterases have previously been described, including enzymes from *Thermotoga maritima*^20^,^21^, *Geobacillus stearothermophilus*^22^,^23^, *Alicyclobacillus acidocaldarius*^24^, *Thermobifida fusca*^25^, *Sulfolobus tokadaii*^26^, *Rhizomucor miehei*^9^, amongst others. To date two esterases and a lipase from *A. fulgidus* have been reported. The AFEST^27^ (locus tag: AF1716) is a member of the hormone-sensitive-lipase family and has a preference for the substrate *p*NP-caprylate. The crystal structure of this enzyme (PDB: 1JJI) revealed several thermophilic adaptations with an increased secondary structure content, reduction in loop extensions and an increase in intramolecular ion pairs compared to a mesophilic homologue^28^. The second esterase Est-AF^29^ (locus tag: AF2336) showed optimal activity towards *p*NP-butyrate and no activity for substrates with a carboxyl group larger than caprylate. This enzyme has been applied in the resolution of ketoprofen ethyl esters. Extensive mutagenesis of Est-AF resulted in an enzyme with improved activity for the production of the *S*-enantiomer of ketoprofen^30^. The biocatalytic synthesis of poly(δ-valerolactone) using AFEST has also recently been demonstrated^31^. The alkaliphilic *A. fulgidus* lipase^32^ (locus tag: AF1763) is also thermostable at 70–90 °C.

Here, we report the biochemical and structural characterisation of a third esterase from the *A. fulgidus* genome, named AF-Est2 (locus tag: AF1537). This belongs to the α/β hydrolase 6 family and the X group of the ESTHER classification. This enzyme was biochemically characterised and shown to be highly thermostable and stable in organic solvents and at extremes of pH. The X-ray structure of the AF-Est2 was determined and reveals the presence of a molecule of Coenzyme A (CoA). This is bound in a unique position in the vicinity of the enzyme active site groove, partially obstructing the alcohol binding pocket. AF-Est2 shows a novel utilisation of CoA where the pantetheine moiety could act as a regulatory function for enzyme activity.

## Results and Discussion

### Substrate specificity

The AF-Est2 enzyme was successfully cloned and over-expressed in *Escherichia coli* and purified using a nickel affinity column and size exclusion chromatography. AF-Est2 was tested against a range of *p*-nitrophenyl (*p*NP) esters and was shown to be active against small to medium acyl chain length esters (C2 to C8), which is consistent with its classification as an esterase rather than a lipase. AF-Est2 showed optimum activity against *p*NP-valerate with a *k*_*cat*_ of 58.9 ± 0.2 s^−1^ and a *K*_*M*_ of 19 ± 1 μM (Table 1). The *k*_*cat*_ values are close for the whole range of acyl ester chain lengths and differences in the catalytic efficiency of the enzyme are due to large differences in the *K*_*M*_ for these different substrates. AF-Est2 was also active against the substrate methyl *p*-toluate which other tested carboxyl esterases are inactive against. Kinetic constants were determined using a phenol red assay giving a *K*_*M*_of 1500 ± 100 μM with *k*_*cat*_ of 1.23 ± 0.07 s^−1^, 0.94 ± 0.02 s^−1^ and 0.08 ± 0.006 s^−1^ at 70 °C, 50 °C and 30 °C respectively. This shows the enzyme is able to accommodate industrially relevant bulky aromatic carboxylate groups.

### Enzyme stability and activity at elevated temperatures

**Figure 1:**
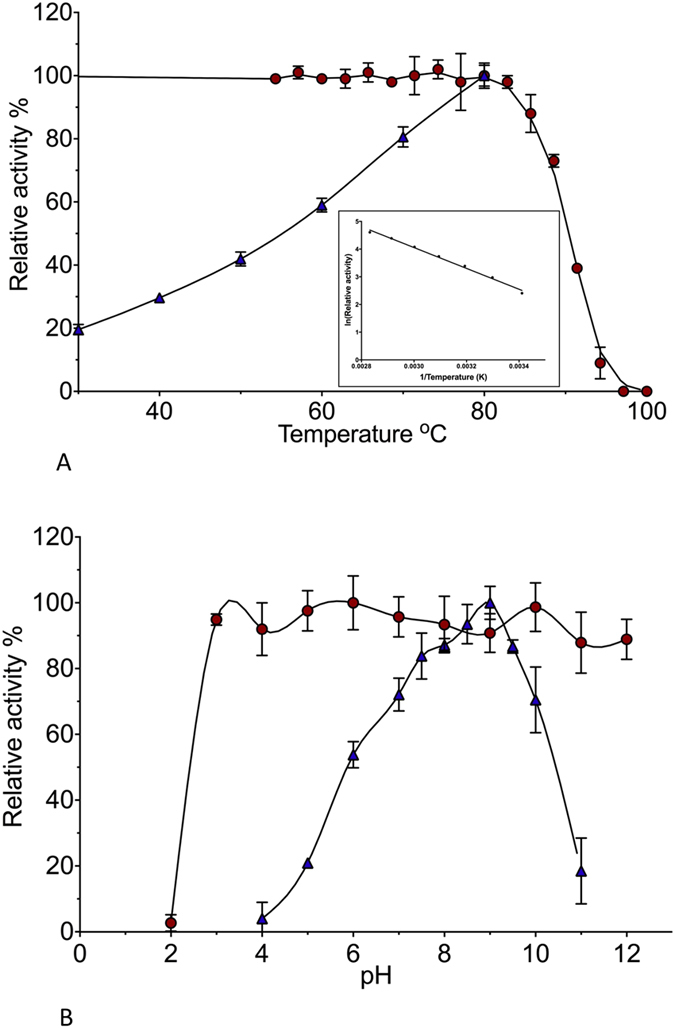
The biochemical characterisation of AF-Est2. (**A**) The effects of temperature on AF-Est2. Blue triangles 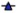 show the relative activity of AF-Est2 with increasing temperature, relative to a maximum activity at 80 °C. Red circles 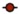 show the relative activity of AF-Est2 incubated for half an hour at various temperatures, against a control kept at room temperature. The insert shows the linearity of the Arrhenius plot of the relative activity with increasing temperature. (**B**) The effects of pH on AF-Est2. Blue triangles 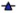 show the relative activity of AF-Est2 at various pH values, relative to its maximum activity at pH 9.0. Red circles 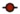 show the activity of AF-Est2 after incubation at various pH values for an hour, before assaying under standard conditions.

To test the temperature tolerance of AF-Est2, it was incubated at elevated temperatures for 30 min and then cooled to room temperature before being assayed. No loss of activity was observed after incubation at temperatures up to 80 °C. This is close to the optimum growth temperature of *A. fulgidus*. As the incubation temperature was increased past this point the enzyme became progressively denatured, retaining no activity after incubation at 95 °C (Fig. 1A). The temperature optimum for activity of AF-Est2 was determined by performing the assay at increasing temperatures. The activity increased with temperature following the Arrhenius equation up to a maximum of 80 °C. A high level of spontaneous substrate hydrolysis prevented measurement of activity beyond 80 °C.

Activity was also measured whilst incubating AF-Est2 at 70 °C, 50 °C and 30 °C for an extended time period. AF-Est2 was able to retain over 50% activity for one week at 70 °C showing the enzyme would be an excellent choice for applications where an elevated temperature may be required for solubilisation of industrial substrates or where enzyme stability under reactions conditions is an issue. At 30 °C and 50 °C an excess of 50% activity remained after 20 days.

### Enzyme stability and activity at different pHs

Features adopted by proteins for stability at high temperatures often allow them to withstand other denaturing conditions such as extremes of pH, presence of organic solvents and proteolysis. We therefore tested the activity of AF-Est2 at pH 7.5 after incubation for one hour at a range of different pH values. No significant change in activity was observed for all incubation pH values between 3.0 and 12.0, showing that the enzyme is stable across an unusually wide range of pH values (Fig. 1B). Incubation at pH 2.0 caused inactivation of the enzyme. Activity assays for AF-Est2 at different pHs showed that the enzyme is active over a broad range of pH values from 5.0 to 11.0 with optimum activity at pH 9.0.

### Solvent stability

The residual activity of the AF-Est2 was tested after incubation in a range of common organic solvents. The enzyme retained activity in nearly all of the solvent conditions after incubation for 1 hour in buffer containing 25 mM Tris-HCl pH 7.5, 100 mM NaCl, and either 10%, 25% or 50% of methanol, ethanol, isopropanol, DMSO, acetonitrile and acetone. Only incubation in 50% isopropanol was significantly detrimental to the enzyme activity resulting in 23% relative activity when compared to the control. As with the stability of AF-Est2 at extremes of pH, adaptions for thermal stability allow the enzyme to withstand the denaturing effects of a range of organic solvents.

### Inhibitors

To assist in determining the mechanism of AF-Est2, the effect of common hydrolase inhibitors on the enzyme was tested. The serine protease inhibitors phenylmethysulfonyl fluoride (PMSF) and benzamidine and the carboxylesterase inhibitor benzil were used in a range of concentrations up to 2 mM. Both reversible inhibitors benzamidine and benzil showed no effect on the reaction rate of AF-Est2 after incubation for half an hour. To fully inhibit AF-Est2, incubation with 100 μM PMSF for 30 min was required. When PMSF was added during the enzymatic reaction a significantly lower inhibition of the enzyme was observed. This would suggest that the substrates or products compete with the PMSF for binding in the active site of the enzyme.

**Figure 2:**
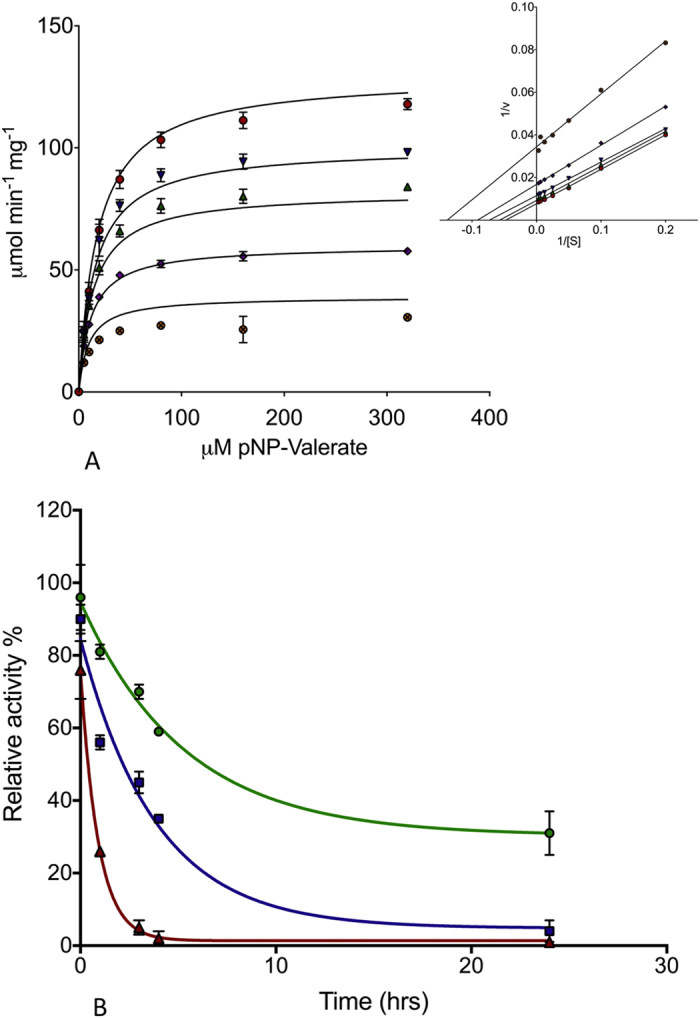
Inhibition of AF-Est2. (**A**) The effect of increasing concentrations of PMSF on the kinetics of AF-Est2 with *p*NP-valerate. Initial rates with varying concentrations of PMSF are displayed as follows: 0 μM PMSF by 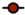, 0.1 μM PMSF by 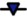, 0.2 μM PMSF by 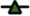, 0.4 μM PMSF by 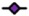 and 0.8 μM PMSF by 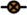. The data has been fitted to the mixed model inhibition equation and the following parameters calculated. *K*_*CAT*_ = 59 ± 0.7 s^−1^, *K*_*M*_ = 18 ± 1 μM, *K*_*I*_ = 1.0 ± 0.3 μM and α = 0.3 ± 0.1 μM. A small alpha value suggests PMSF acts more as an uncompetitive inhibitor, which is reflected when the data is shown as a Lineweaver-Burk plot. (**B**) The relative activity of AF-Est2 after incubation with various concentrations of diamide over time, shown as 100 mM 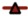, 10 mM 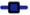 and 1 mM 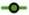. The activity is relative to a control sample with no diamide. Error bars show the standard error of three replicates.

Investigating the kinetics of the enzyme inhibition with different concentrations of PMSF (Fig. 2A) revealed the mode of inhibition to be mixed, with a *K*_*I*_ of 1.0 ± 0.3 μM and an Alpha value of 0.3 ± 0.1. A small Alpha value suggests PMSF acts more as an uncompetitive inhibitor than a competitive inhibitor, again suggesting an unusual mode of inhibition for this enzyme^33^.

### Structural determination

AF-Est2 was crystallized in the space group *P*2_1_2_1_2_1_ with cell dimensions of a = 55.6 Å, b = 67.6 Å, c = 139.5 Å, α=β=γ=90°. The asymmetric unit contains two protein monomers each with a molecular mass of 28.1 kDa giving a solvent content of 46%. The structure has been isotropically refined to a resolution of 1.4 Å with a final *R*_*free*_ of 17.5 (Table 2). Several data sets were collected in an attempt to get protein-ligand complexes from crystals soaked or co-crystallised with *p*NP esters, benzyl, benzamidine and PMSF. Data for putative *p*NP-valerate and PMSF complexes were collected to high resolution, but contained no density for the bound ligand.

**Figure 3:**
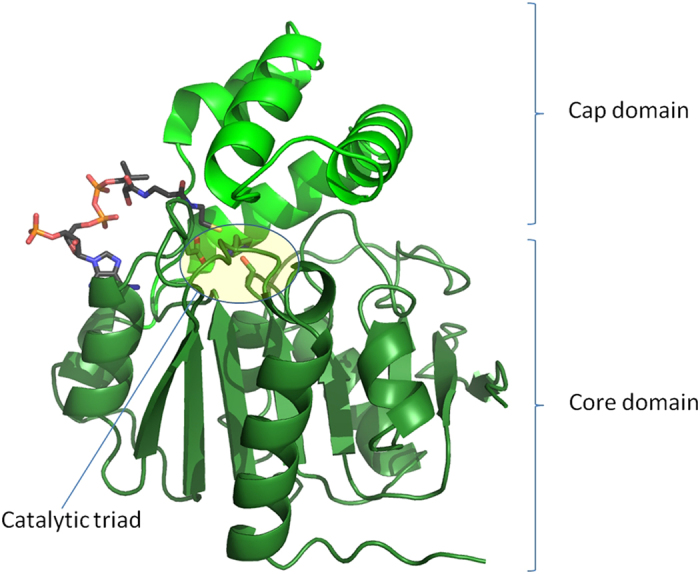
A cartoon representation of the AF-Est2 structure showing the core and cap domains in dark green and light green respectively, with the bound CoA molecule shown as a stick model. The catalytic triad residues are shown as stick models and the active site is highlighted as a yellow box. Figures 3, 4a,c, 5 and 6 were prepared using PyMol Molecular Graphics System (Schrödinger LLC).

The structure reveals, as expected, a canonical α/β hydrolase fold core domain (Asp1-Arg117 and Asp176-His254), and a cap domain (Leu118-Phe175) formed by helices α5-α8 (Fig. 3). Six helices of the core domain surround an eight stranded β-sheet of mixed type with connectivity 1,2,−1x,2x,1x,1x,1x with direction +−++++++. The catalytic Ser89 residue in both molecules of AF-Est2 is an outlier on the Ramachandran plot. The strained conformation of the catalytic serine residue is observed in the structures of most other α/β hydrolase fold enzymes^34^. There are no residues in a *cis* conformation in AF-Est2. The structure also contains two CoA molecules at full occupancy, several ordered PEG molecules and a single citrate molecule bound at a crystal contact. This citrate ligand originates from the crystallisation solution and could have contributed to the formation of more ordered crystals.

Although the asymmetric unit of AF-Est2 contains two monomers, these do not form oligomers in the crystal. This is in agreement with the chromatography results where the protein eluted as a monomer from a calibrated size exclusion column.

### Ligand assignment

**Figure 4:**
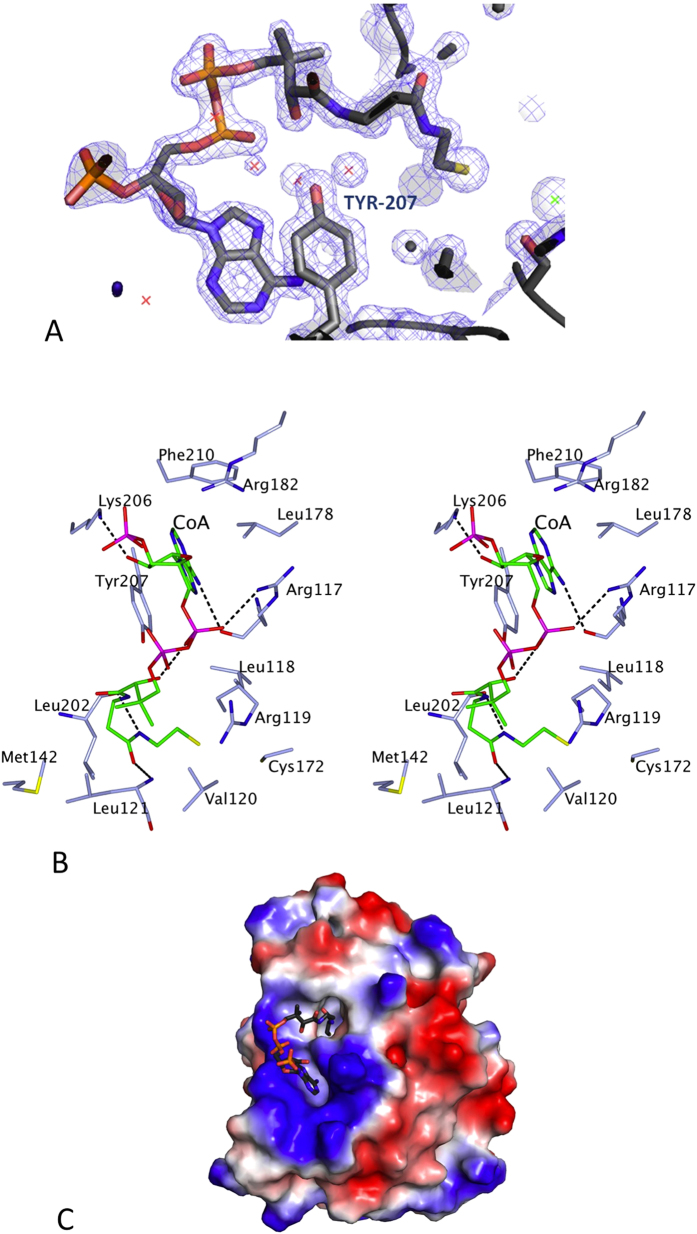
The interactions of the CoA ligand bound to AF-Est2. (**A**) A stereo diagram showing the electron density maps in the region of the CoA binding to AF-Est2. The 2F_o_ − F_c_ (blue) is contoured at 1.2 σ and the F_o_ − F_c_ map is contoured at 3.5 σ (green) and −3.2 σ (red). The ligand and amino acid residues are shown as stick models. Solvent molecules are shown as red crosses, a Cl^−^ ion is shown as a green cross. (**B**) A stereo diagram showing the CoA binding site of AF-Est2. The CoA molecule is shown as a stick model with carbon atoms coloured in green. Amino acid side chains of residues implicated in the ligand binding are coloured in light blue with hydrogen bonds shown as black dashes. The Figure 4b was prepared using CCP4 mg^58^. (**C**) The electrostatic potential surface of the AF-Est2 CoA complex. The positive charge is shown in blue and the negative charge is shown in red. The ligand molecule was not used in the surface calculation and is shown as a stick model.

When most of the AF-Est2 model was built using the original 2.1 Å resolution data, a significant stretch of continuous density became apparent on both the 2*F*_*o*_ − *F*_*c*_ and the *F*_*o*_ − *F*_*c*_ maps in the proximity of the AF-Est2 catalytic triad in both monomers. Modelling of a bound polypeptide did not produce a convincing match to the observed density. A molecule of CoA provided the best fit for the un-assigned density. Refinement at the higher resolution of 1.4 Å confirmed the full occupancy of this CoA ligand in the AF-Est2 structure (Fig. 4A).

### The CoA binding groove

The pantetheine group of the CoA points directly into the active site. The remainder of the CoA molecule is bound in a groove on the surface of the enzyme (Fig. 4B,C). This groove is formed by the loop region consisting of residues 116–121, preceding α5 on one side and the loop region of residues 202–207 which precedes α9 on the other side. There is an ion pair formed by the diphosphate moiety of the CoA and the guanidinium group of Arg117. The residues Arg119 and Arg182 could also form ion pairs with the diphosphate and phosphate groups of CoA, respectively, but do not do so in this structure. The CoA also forms several specific H-bonds and hydrophobic interactions. The adenine ring of CoA is stacked in a pocket between the side chains of Tyr207 and Leu178 (Fig. 4B) and its N6A atom makes a H-bond to the main chain oxygen of Arg117. The side chain of Lys206 is H-bonded to O2B of the ribose ring of CoA. The N4P and O5P of the pantetheine group of the CoA are H-bonded to main chain oxygen of Leu202 and the main chain nitrogen of Leu121, respectively. It appears that the CoA has a high affinity for the enzyme and is tightly bound since no CoA was added during expression or purification of the enzyme. Extensive dialysis of the purified enzyme did not remove the CoA molecule which may contribute to the high thermostability observed for this enzyme.

The NCBI reference sequence database contains about twenty protein sequences from both archaeal and bacterial sources with higher than 35% sequence identity to AF-Est2 over more than 90% of its length. Residues that are involved in CoA binding in AF-Est2 are either conserved or conservatively replaced in the majority of these enzymes. This suggests that these other enzymes could also bind CoA, however, in the absence of their biochemical or structural characterization this cannot be determined. The structure of the AF-Est2 described in this paper is the first example to show the binding of CoA to an esterase enzyme in this conformation.

Since the AF-Est2 binds CoA this enzyme could have a role in reversible hydrolysis of CoA esters. However, no activity was observed towards the common CoA esters, acetyl-CoA and succinyl-CoA (data not shown) confirming that AF-Est2 does not function as a thioesterase. If CoA esters were a substrate for AF-Est2 they would have moderate affinity for the enzyme to allow the rapid dissociation of the product CoA which would not bind as tightly to the enzyme as observed in these studies. It therefore appears that the tightly bound CoA acts as an integral part of the enzyme.

### Active site

The active site is located at the interface of the core and the cap domains with the conserved catalytic triad composed of Ser89, His228 and Asp200. The catalytic serine is in a strained conformation and is located in a tight nucleophilic elbow at the end of strand β5 with the conserved esterase signature sequence Gly-His-Ser-Leu-Gly.

A comparison of the structure of AF-Est2 with structures of the ligand complexes of other proteins of the α/β hydrolase family 6, allows prediction of its active site pockets. The carboxyl binding pocket of AF-Est2 is likely to be formed by the loop region between β3 and α1, and helices α5, α6 and α7. The side chains of residues Met229, Lys154, Phe158, Leu140, Met139 and Ser32, with Phe158 and Lys154, are responsible for defining the size of the carboxyl ester group to be hydrolysed. This largely hydrophobic pocket defines the enzyme’s optimal activity towards *p*NP-valerate (Table 1). The residues Lys154 and Phe158 (helix α7), at the far end of the carboxyl binding pocket, restrict the chain length of the substrate that can be hydrolysed. It appears that movement of these side chains would be required for the binding of the caprylate chain, which would explain the lower levels of affinity of AF-Est2 towards this *p*NP-ester (Table 1). The enzyme’s affinity towards the *p*NP-acetate and *p*NP-propionate is also relatively low, which appears to be due to the reduced binding interactions available in the active site for these substrates.

### CoA obstructs the alcohol binding pocket of the active site

The hydrophobic alcohol pocket is formed by the side chains of residues Leu118, Val120, Leu169 and Cys172, and is partially obstructed by the pantetheine group of the CoA. The alcohol pocket is responsible for binding the *p*NP moiety of the associated substrates. The predicted binding of the *p*NP moiety is thought to be provided by the main chain region of helix α6 between the side chains of Val120 and Leu169. For successful *p*NP ester hydrolysis, displacement of the pantetheine group of the CoA would be required, since modelling has shown it to be a major steric hindrance for the binding of the relatively large *p*NP group in the alcohol pocket. However high activity of AF-Est2 towards the *p*NP esters suggests that the flexible CoA pantetheine group is displaced from the alcohol pocket upon substrate binding.

### Thiol oxidation inhibits AF-Est2 activity towards *p*NP esters

The thiol group of CoA is located around 4 Å away from the thiol group of Cys172 in AF-Est2 and would form a disulfide bond with Cys172. The necessity of the CoA pantetheine group displacement for *p*NP ester hydrolysis was confirmed by incubation of the AF-Est2 with the disulfide inducing agent diamide which enforces the formation of a covalent bond between the thiol groups of CoA and Cys172 (Fig. 2B). This oxidised enzyme shows no activity towards *p*NP-valerate demonstrating that this enzyme is inhibited by the formation of the disulfide. Modelling studies suggest that hydrolysis of substrate esters with small alcohol groups (methyl or ethyl) may not be inhibited by the disulfide formation.

Since the AF-Est2 does not have a signal sequence it is assumed to be a cytosolic protein, with reduced disulfides. It would appear that the CoA ligand may control the levels of activity of AF-Est2 towards carboxyl esters since activity towards esters with larger alcohol groups will be reduced due to the partial obstruction of the active site by the CoA. It is possible that AF-Est2 could have evolved from an ancestral protein with CoA thioesterase activity.

### Comparison of AF-Est2 with related enzymes of the α/β hydrolase family

The AF-Est2 belongs to the α/β hydrolase family 6 in the Pfam classification^35^. The enzymes of this family are reported to catalyse a variety of different reactions. Examples include a *Pseudomonas fluorescens* esterase^36^, bacterial non-heme haloperoxidases^37^, the *Burkholderia xenovorans* 3-oxodipate enol lactonase^38^ and *Aureobacterium* (−) γ-lactamase^39^. Depending on the features of the active site and the type of reaction that is catalysed, some of the enzymes have esterase activity.

**Figure 5:**
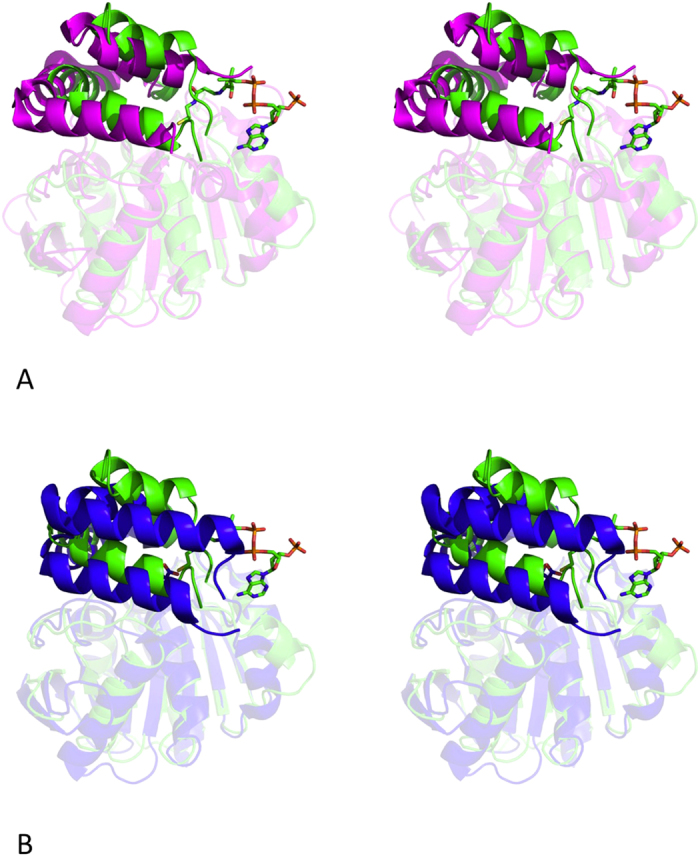
A comparison of the overall fold of AF-Est2 with that of related proteins. (**A**) A stereo diagram showing the superposition of AF-Est2 (green) with the open form of Hsad (magenta; PDB 2VF2). The core domains are shown as transparent cartoon models and the cap domains are shown as full colour cartoon models. The AF-Est2 cap domain restrains the size of the ligand capable of binding to the enzyme active site. The CoA ligand is shown as a stick model. (**B**) A stereo diagram showing the superposition of AF-Est2 (green) with the malate complex of the TtEst (PDB 4UHE). The core domains are shown as transparent cartoon models and the cap domains are shown as full colour cartoon models. The relative positions of the core and cap domains are more similar between AF-Est2 and TtEst, than between AF-Est2 and Hsad. The ligands CoA and the malate which maps the alcohol binding pocket in TtEst are shown as stick models.

The closest sequence homologues of AF-Est2 in the structural database are the C-C bond hydrolases such as the tetrameric Mhcp^40^ from *E. coli* (PDB 1U2E) and the tetrameric meta-cleavage product (MCP) hydrolase (Hsad) from *Mycobacterium tuberculosis*^41^ (PDB 2VF2). The latter enzyme (with only 27% identity to AF-Est2 over 95% of the amino acid sequence) catalyses the hydrolytic cleavage of a C-C bond in an intermediate of cholesterol metabolism and is also capable of catalysing ester bond hydrolysis *in vitro*^41^. The active site Ser114Ala mutation of Hsad has allowed elucidation of the conformational changes of the enzyme structure which accompany the substrate binding^42^ (PDB 2WUF). The relative positions of the cap and core domains differ between Hsad and AF-Est2 which causes a shift of the helix α8 by 6.5 Å towards the catalytic triad in AF-Est2 (Fig. 5A). The helices α5, α6 and α7 of the cap domain of AF-Est2 are shifted by approximately 3 Å in relation to their equivalent helices in the Hsad structure. The loop region between the core and the cap domain is involved in the CoA binding in AF-Est2. This loop is significantly longer in Hsad and occupies the space of the CoA groove in AF-Est2. The differences of the cap domain positions would prevent the binding of the MCP substrates to AF-Est2. In Hsad, the active site entrance on the interface of the domains is open in the absence of ligand and partially closes in its presence^42^. However, it is much more closed in AF-Est2, even when the obstructing CoA molecule is not taken into account. The shorter loop region between helices α8 and α9 in AF-Est2 would restrict the relative secondary structure movements observed in the Hsad enzyme.

The MCP hydrolases have two conserved sub-sites: a hydrophilic polar sub-site binding a substrate dienoate moiety, and a non-polar sub-site binding the hydrophobic part of the molecule. The different position of helix α8 and the loops connecting the core and the cap domains would not allow AF-Est2 to form such a non-polar binding site. The polar binding site responsible for MCP hydrolase activity also shows low conservation of both the positioning of the main chain backbone and the amino acid sequence in this region. Therefore, it is not expected that AF-Est2 would have C-C hydrolase activity.

AF-Est2 also shares 27% identity (over 79% of the amino acid sequence) with the monomeric *E. coli* BioH carboxyl esterase^43^ (PDB 1M33), an enzyme involved in biotin biosynthesis. The enzyme has a preference for carboxyl esterase activity towards short acyl chain substrates and has weak thioesterase activity^43^. Whilst the core domains of the two enzymes superimpose closely, there is a relative movement of the helices α5-α8 that comprise the cap domain. The relative movement of the core and the cap domains between the model and the target could be the reason why an MR solution could not be found for AF-Est2 using Hsad and BioH as models, despite their greater sequence similarity.

AF-Est2 and BioH display little structural conservation in the active site region. One of the two helices in BioH which define the carboxyl pocket (α5) is displaced 5 Å away from the active site in AF-Est2 and the other (α6), which defines the far edge of the pocket, is in a different orientation. BioH shows optimal activity towards *p*NP-acetate and *p*NP-propionate with lower activities observed for *p*NP-butyrate and *p*NP-caprylate. In contrast, AF-Est2 shows a similar activity towards acetate and a much greater activity towards *p*NP-propionate and optimal activity towards *p*NP-valerate. The side chains of Gln147 (equivalent Ala143 in AF-Est2), Trp81 (His88) and Leu24 (Ser32) give BioH a more restrictive carboxyl binding pocket compared to AF-Est2, thereby explaining the difference in substrate specificity between the two enzymes.

Using the Dali lite server^44^, AF-Est2 shows best structural alignment to an enol lactonase^38^ (RMSD 2.2 Å; PDB 2XUA) and a carboxyl esterase from a newly isolated thermophilic Planctomyces species, *Thermogutta terrifontis* (TtEst; RMSD 2.5 Å) recently described by our group^45^. The core domains between TtEst and AF-Est2 superimpose closely and the relative positions of the core and the cap domains are similar (Fig. 5B). However, helices α5 and α8 are displaced in AF-Est2 compared to the TtEst.

The carboxyl binding pocket in AF-Est2 occupies a larger volume than that in TtEst. This is consistent with AF-Est2′s preference towards larger butyrate and valerate esters for which the TtEst shows limited activity^45^. Mutagenesis studies in TtEst have confirmed that Leu37 hinders the binding of esters with side chains larger than propionate. The corresponding Ser32 in AF-Est2 allows binding of the larger carboxyl side chains. Although most of the residues in the carboxyl pocket are not conserved between AF-Est2 and TtEst, they both are predominantly hydrophobic.

The 3-oxodipate enol lactonase and (−) γ-lactamase, which both are active only towards *p*NP-acetate, have bulky Trp side chains obstructing the binding of the longer chain substrates in the carboxyl pocket.

The alcohol pocket in AF-Est2 is significantly smaller than that of TtEst and the 3-oxodipate enol lactonase. This is due to obstruction by the panthotheine group of the CoA molecule and to the different position of helix α5.

### Comparison of CoA binding with another α/β hydrolase fold thioesterase

**Figure 6:**
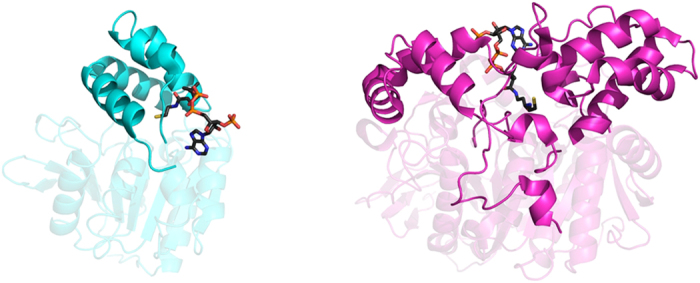
A comparison of the CoA binding to both AF-Est2 (cyan) and human carboxylesterase 1 (magenta) shown as cartoon models in the same orientation. The core domains are shown as transparent models and the cap domains are shown in full colour. The CoA ligands bound to the two proteins are shown as ball-and-stick models with carbon atoms coloured in grey. The CoA molecules bind to different parts of the core domain in these two proteins and their panthetheine groups come into the enzyme active site from the different directions.

Since the results presented in this paper suggest that the AF-Est2 has inherited the CoA binding from an ancestral protein with thioesterase activity, it is interesting to compare this ligand binding in AF-Est2 with other proteins of the α/β hydrolase fold family. Since only a relatively small proportion of CoA thioesterases have the α/β hydrolase fold, a search of the PDB revealed a single enzyme of this family which also binds CoA. This human carboxyl esterase 1 (PDB 2DQZ and 2H7C) is involved in cholesterol ester hydrolysis, fatty acyl CoA hydrolysis, acyl-CoA:cholesterol acyl transfer, and fatty acyl ethyl ester synthesis^46^. The monomer of the hexameric human enzyme is twice as large as AF-Est2; however, the core domains of the two proteins superimpose well. In contrast, the cap domain of the human esterase has no similarity to the cap domain of AF-Est2 and contains three distinct ligand binding sites. The mode of binding of the CoA ligand to one of these sites in the human carboxyl esterase shares little similarity to that observed in AF-Est2 (Fig. 6). In AF-Est2, the CoA molecule is bound in the groove on the surface between the C-terminal part of the core domain and the cap domain. In the human carboxyl esterase the CoA binds between the N-terminal part of the core domain and the extended cap domain. This results in the pantetheine groups of CoA coming into the enzyme active sites of AF-Est2 and human carboxyl esterase 1 from opposite directions. These differences confirm that AF-Est2 shows an entirely novel form of CoA binding and suggests that this enzyme has evolved to utilise the pantetheine group of CoA as an integral part of its active site.

## Conclusions

The AF-Est2 esterase structure has shown a novel binding mode for a CoA molecule which is located in a groove overlapping the active site of the enzyme where the pantetheine moiety is partially obstructing the alcohol binding pocket. This suggests that the CoA may have a role in reducing activity towards esters with a large alcohol group. The AF-Est2 does not appear to be related to the α/β hydrolase fold eukaryotic carboxyl esterases that also bind CoA. The structures of AF-Est2 and human carboxyl esterase reveal that the pantetheine group approaches the active site from opposite directions between the two enzymes. The AF-Est2 enzyme is so far unique in its mode of binding of CoA and could represent the first example of a new group of esterases. The AF-Est2 reported here shows no thioesterase activity despite the fact that it could have evolved from an ancestral protein with CoA thioesterase activity.

The detailed biochemical and structural comparisons of AF-Est2 with other related Pfam family 6 α/β hydrolase enzymes as reported in this paper is of general interest from the aspect of natural enzyme evolution which has occurred within this large enzyme family.

The AF-Est2 esterase from the thermophilic archaeon *Archaeoglobus* has potential applications in industrial biocatalysis due to its overall stability to high temperature, a broad pH range and tolerance to organic solvents.

## Methods

### Expression and purification

The gene encoding AF-Est2 (locus tag: AF1537; Uniprot accession number: O28735) was PCR-amplified, without its stop codon, using chromosomal DNA of *A. fulgidus* as a template and the two primers 5′-GCGCCATGGACCTGGAGAGAGTATTCATCG-3′ and 5′-GGGCTCGAGAACCCCAACTTTTTTGAGAAACTTTTCAAGCGC-3′, introducing respectively a *Nco*I and *Xho*I restriction site. The generated PCR product was digested by *Nco*I and *Xho*I and the product was purified and ligated into the protein expression vector pET24d (EMD Millipore) digested with the same restriction enzymes, resulting in the plasmid pWUR365 for the expression of the C-terminal 6x-His-tag AF-Est2 protein.

The pWUR365 vector was transformed into the *E. coli* strain BL21-CodonPlus (DE3)-RIPL (Agilent) cells which were grown on Luria-Bertani (LB) medium containing 50 μg/ml each of kanamycin, chloramphenicol and streptomycin for protein expression. Cultures were grown at 37 °C, 225 rpm until approximately OD_600_ 0.6, at which point protein expression was induced by the addition of 1 mM IPTG. The cultures were left shaking at 30 °C for a further 18 hours. Cells were harvested by centrifugation and resuspended in 20 mM Tris-HCl pH 8.0, 10 mM imidazole. Cell lysis was achieved by sonication on ice followed by centrifugation. AF-Est2 was purified using a 1 ml His-Trap FF crude column (GE Healthcare) using an elution gradient from 20 to 500 mM imidazole in 20 mM Tris-HCl pH 8.0, 0.5 M NaCl. The enzyme was then applied to a calibrated Superdex 200 HiLoad 16/60 size exclusion column (GE Healthcare) and was eluted with one column volume of a buffer of 25 mM Tris-HCl, pH 7.5, 0.1 M NaCl.

### Standard assays

Assays were carried out using *p*NP ester substrates as previously described^47^. A 50 mM stock of each substrate in DMSO was prepared and stored at −80 °C until required. Unless otherwise specified, each reaction was performed in assay buffer containing 50 mM Tris-HCl pH 7.5, 1 mM substrate, 0.125 μg/ml of enzyme and carried out in triplicate in standard 96-well microplates (Greiner 655101). Reactions were started by the addition of substrate after pre-incubation at 30 °C for 5 min, and monitored by following the absorbance at 405 nm over 10 min in a Tecan Infinite M200 PRO plate reader. All activity measurements included a reading for the blank rate of hydrolysis which was subtracted from the observed activity, compensating for any autohydrolysis of the *p*NP esters.

The molar extinction coefficient of *p*NP was determined for every condition prior to measurements. One Unit of esterase activity was defined as the amount of protein releasing 1 μmol/min of *p*NP.

To measure the CoA thioesterase activity a DTNB-based assay^48^, which measures the level of free thiols (and hence formed CoA) was performed following incubation of the enzyme with acetyl-CoA and succinyl-CoA.

### Substrate specificity and kinetics

Kinetic analysis of substrate specificity was carried out by measuring the activity of AF-Est2 against a range of *p*NP esters (C2 to C12 and *p*NP-benzoate) over a range of ten substrate concentrations from 5 to 2500 μM. The initial reaction rates were fitted to the Michaelis-Menten equation by non-linear least squares regression using GraphPad Prism 5.0.

Phenol red was used to monitor the change in pH as a result of the hydrolysis of the industrial substrate methyl *p*-toluate by the change in absorbance at 540 nM. The 1 ml assay was prepared using 20 μg/ml phenol red, 1 mM HEPES pH 8.0 at each assay temperature. A spectrophotometer with a Peltier temperature controller was used for assays at 50 °C and 70 °C with 13.6 μg/ml of enzyme. Assays at 30 °C were carried out in a standard 96-well microplate reader in a 200 μl volume with 25.0 μg/ml of enzyme. All assays were carried out for 10 min after addition of the substrate. A standard curve from 0 to 300 μM of *p*-toluic acid was generated for each assay temperature. Activity was measured at eight concentrations of methyl *p*-toluate between 0 and 5000 μM and the initial reaction rates were fitted as described above.

### Effects of temperature

The thermostability of AF-Est2 was assessed by incubating samples at a range of temperatures for half an hour using the temperature gradient function of a T100 thermocycler (Bio-Rad). Following incubation samples were cooled to 4 °C before assaying activity against *p*NP-valerate, relative to a control sample kept at room temperature.

To examine the effect of temperature on the activity of AF-Est2, a standard assay buffer was prepared and titrated to the correct pH at temperatures from 20 °C to 80 °C. Using an Evolution 300UV-Vis spectrophotometer (Thermo Scientific) with a Peltier heater, 970 μl of assay buffer was equilibrated to temperature in a 1 ml quartz cuvette. The blank rate with 0.5 mM *p*NP-valerate was measured before the addition of 0.125 μg of enzyme. A 10 min continuous assay was performed and the data analysed to use only initial linear rates.

To measure the stability of AF-Est2 at 70 °C, 50 °C and 30 °C aliquots of AF-Est2 were prepared at 1 mg/ml in 25 mM HEPES pH 7.5, 100 mM NaCl and 0.01% sodium azide. Every few days 0.2 μg of enzyme was taken from each aliquot was used to measure activity against 0.5 mM *p*NP-valerate in triplicate, with rates calculated relative to those at the beginning of the experiment.

### Effects of pH

To test the stability of AF-Est2 at various pHs, AF-Est2 was incubated for one hour at room temperature in the following buffers covering a range of pH values from 2 to 12: 100 mM KCl-HCl pH 2.0, 100 mM glycine-HCl pH 3.0, 100 mM sodium acetate pH 4.0 and pH 5.0, 100 mM sodium phosphate pH 6.0, 100 mM Tris-HCl pH 7.0, pH 8.0 and pH 9.0, 100 mM glycine-NaOH pH 10.0, 100 mM sodium dihydrogen orthophosphate-NaOH pH 11.0 and pH 12.0. The enzyme was then diluted 250 fold into 50 mM Tris-HCl pH 7.5, to a final concentration of 2.5 μg/ml, and assayed against *p*NP-caprylate (C8).

To access the effect of pH on enzyme activity, reactions were carried out using the buffers described above in place of the standard reaction buffer. The *p*NP-caprylate was used as the substrate to reduce spontaneous hydrolysis at alkaline pHs. Readings were taken at 348 nm, the isobestic point of *p*NP, to remove the effect of pH on the readings.

### Effect of solvents

To examine the stability of AF-Est2 in various solvents, the enzyme was incubated for 1 hour in buffer containing 25 mM Tris-HCl pH 7.5, 100 mM NaCl, and either 10%, 25% or 50% solvent. Methanol, ethanol, isopropanol, DMSO, acetonitrile and acetone were tested. A control sample was incubated with no solvent to compare relative activity. Samples were then diluted 1 in 2000 in assay buffer to measure enzyme activity.

### Effect of inhibitors

The inhibitors PMSF, benzamidine and benzil at concentrations in the 0–2 mM range were incubated with enzyme at 9 nM for 30 min before measuring enzyme activity by the addition of the substrate, 0.5 mM *p*NP-valerate.

The mode of PMSF inhibition was determined using Michaelis-Menten kinetics with eight different *p*NP-valerate concentrations in the presence of five inhibitor concentrations. The best equation to describe the inhibition data was selected by fitting a non-linear least squares regression in GraphPad Prism 5.0.

### Diamide treatment

0.25 mg/ml enzyme was incubated in a 1, 10 or 100 mM diamide, 25 mM Tris-HCl pH 7.5, 100 mM NaCl solution for 1 hour before a 1 in 250 dilution into 25 mM Tris-HCl pH 7.5, 100 mM NaCl was made. A control incubation was also performed without the addition of diamide. Enzyme assays were performed as specified previously against *p*NP-valerate. Activity was calculated as relative to the control sample.

### Crystallization

AF-Est2 was concentrated to ~12 mg/ml using a 10 kDa membrane Vivaspin (Vivaproducts) and microbatch crystallization trials were set up using an Oryx6 crystallization robot (Douglas Instruments) using the JCSG Screen+™ (Molecular Dimensions). The droplet contained a 50:50 ratio of protein solution to screen and was covered with Al’s oil (50:50 mix of silicon and paraffin oils) before being stored at 20 °C.

Native crystals appeared within one week in 100 mM ammonium chloride and 10% (w/v) PEG3350 and were cooled in liquid N_2_ using a cryoprotectant consisting of 100 mM ammonium chloride, 10% (w/v) PEG3350, 35% (v/v) PEG400, 25 mM Tris pH 7.5 and 100 mM NaCl. Multiple attempts of inhibitor and substrate co-crystallisation produced crystals in conditions related to those of native crystals. Additionally, native crystals were soaked for 30–300 sec in the cryoprotectant containing variable concentrations of ligands.

### X-ray data collection and structure solution

Native AF-Est2 data were collected to 2.1 Å and later to 1.4 Å on beamline I03, at the Diamond Synchrotron light source (Didcot, UK) at 100 K in a stream of gaseous nitrogen using a Pilatus detector (Dectris). Data were processed and scaled using XDS^49^ and AIMLESS^50^ in the Xia2^51^ pipeline. All further data and model manipulation was carried out using the CCP4 suite of programs^52^.

Phases for the initial structure were determined using these 2.1 Å resolution data by the molecular replacement method implemented in the MORDA pipeline (Vagin and Lebedev; http://www.biomexsolutions.co.uk/morda). The best solution was found with the bromoperoxidase A1 monomeric model^37^ (PDB 1A8Q) which shares 27% sequence identity to AF-Est2. Two monomers of the search model were positioned in the AF-Est2 unit cell with a Z-score of 9.6 and in the course of initial refinement *R*_*free*_ was reduced from 57.6% to 51.9%. MORDA has estimated the probability of the resulting solution to be correct at 81%.

The phases were improved by two fold NCS averaging using DM^53^. The averaged phases were used in phased refinement implemented in the REFMAC5^54^. The combined partial model and averaged phases produced a good quality map that allowed re-building of AF-Est2 structure in COOT^55^. The unknown ligand was identified as CoA at this stage.

Refinement with both anisotropic and isotropic B-factors was attempted since for 1.4 Å the ratio of independent reflections to refined atoms (including alternative conformations) is 20, which is a borderline case for anisotropic B-factor refinement according to PDB REDO^56^. After the map inspection isotropic B-factor refinement was selected.

The statistics of the data processing and parameters of the final refined models are given in Table 2. The dictionary definitions for *p*NP ester ligands used for docking were prepared using JLIGAND^57^ and the manual docking of ligands was performed in COOT.

## Additional Information

**Accession codes:** The coordinates and structure factors for AF-Est2 have been deposited in the Protein Data Bank with code: 5FRD.

**How to cite this article**: Sayer, C. *et al*. Structural and biochemical characterisation of *Archaeoglobus fulgidus* esterase reveals a bound CoA molecule in the vicinity of the active site. *Sci. Rep*. **6**, 25542; doi: 10.1038/srep25542 (2016).

## Figures and Tables

**Table 1:** The kinetic characterisation of AF-Est2 using *p*NP-esters with varying acyl chain length and *p*NP-benzoate as substrates.

	*k*_*cat*_ (s^−1^)	K_*M*_ (μM)	*k*_*cat*_*/*K _*M*_ (s^−1^ μM^−1^)
C2	21.5 ± 0.2	620 ± 10	0.0349 ± 0.0007
C3	47.8 ± 0.5	254 ± 8	0.189 ± 0.006
C4	55.5 ± 0.5	92 ± 3	0.61 ± 0.02
C5	58.9 ± 0.2	19 ± 1	3.1 ± 0.2
C8	46.0 ± 0.3	120 ± 10	0.37 ± 0.03
C12	0	N/A	0
Benzoate	0	N/A	0

No activity was detected for substrates with an acyl chain length of twelve carbons or more.

**Table 2:** The AF-Est2 data processing and structural refinement statistics.

Crystal	COA complex
Beamline (Diamond)	I03
Resolution (Å)	70.29-1.40 (1.44-1.40)^a^
Wavelength (Å)	0.9763
Space group	*P*2_1_2_1_2_1_
Cell dimensions	a, b, c = 55.1, 67.2, 140.6 Å; α, β, γ = 90°
No. of protomers in A.U.	2
Solvent content (%); V_M_ (Å^3^ Da^−1^)	46.4; 2.31
Unique reflections	102188
Redundancy	5.6 (2.5)
Completeness	98.9 (89.6)
<(I)/ σ (I)>	18.9 (2.0)
R_sym_ (%)	4.4 (46.1)
Wilson B factor (Å^2^)	24.5
Overall R-factor (%)	15.6
R_free_ (5% total data) %	17.3
Residues refined	508
No. of waters modelled	514
RMSD bond length (Å)	0.011 [0.019]^b^
RMSD bond angle (°)	1.6 [2.0]
Occupancy of ligand	1.0
Average B factor	
Protein (Å^2^)	22.8
Water (Å^2^)	35.5
Ligand	24.8
Ramachandran analysis (% of residues)	
Most favoured	88.7
Additionally allowed	10.9
Generously allowed	0.0
Disallowed	0.4
G-factor	0.1

^a^Values for the outer resolution shell are given in parentheses.

^b^Target values are given in brackets. *R*_*sym*_ = ∑_h_∑_J_| < I_h_ > −I_J_(h) |/∑_h_∑_J_ I_J_(h), where I(h) is the intensity of the reflections h, ∑_h_ is the sum over all the reflections and ∑_J_ is the sum over J measurements of the reflections. The individual atomic B-factors were refined isotropically. *R*_*cryst*_ = ∑||F_o_| − |F_c_||/∑|F_o_|. Wilson B-factor was estimated by SFCHECK^59^. The Ramachandran plot analysis and G-factor calculation were performed by PROCHECK^60^.
